# Supplementary material for: Rhinovirus/enterovirus was the most common respiratory virus detected in adults with severe acute respiratory infections pre-COVID-19 in Kuala Lumpur, Malaysia
Source: PLoS One. 2022 Sep 2;17(9):e0273697. doi: 10.1371/journal.pone.0273697 (PMC9439195; doi:10.1371/journal.pone.0273697)
Supplement: S1 Data — (DOCX) [file pone.0273697.s001.docx]

**S1 Data.** Validation of molecular assays.

**Methods**

RT-qPCR assays for MERS-CoV^1^ and SARS-CoV^2^ and qPCR assays for bacterial select agents (*Burkholderia pseudomallei*,^3^ *Coxiella burnetii*,^4^ *Bacillus anthracis*,^5^ *Francisella tularensis*,^6^ and *Yersinia pestis*^7^ were validated using published protocols with minor modifications in the primer and probes sequences as listed in Table 1. Briefly, RT-qPCR assay for MERS-CoV and SARS-CoV was performed in a 10µl reaction consisting Taqman Fast Virus 1-step master mix (Applied Biosystem, USA), primers, probe, nuclease-free water and 2.5µl of nucleic acid. The thermocycling conditions were as follows: 50°C for 5min, 95°C for 20s, followed by 40 cycles of 95°C for 3s and 60°C for 30s. The qPCR assay for bacterial select agents were performed using Taqman Fast Advanced master mix (Applied Biosystem, USA), primers and probe, nuclease-free water and 1µl of nucleic acid to a final volume of 10µl. The reaction mixes were subjected to 50°C for 2min, 95°C for 20s, followed by 40 cycles of 95°C for 1s and 60°C for 20s. The standard curve was generated for each molecular assay to determine the regression coefficient (R^2^), amplification efficiency and limit of detection (LoD) using serial 10-fold dilution (10^6^ to 1 copies/reaction) of standard control.

**Table 1.** Primers and probes used for RT-qPCR and qPCR assays.

| Respiratory pathogens | Target | Sequence (5’ to 3’) | PCR product size (bp) | Reference |
| --- | --- | --- | --- | --- |
| MERS-CoV | upE | Forward: GCAACGCGCGATTCAGTT  Reverse: GCCTCTACACGGGACCCATA  Probe: (FAM)CTCTTCACATAATCGCCCCGAGCTCG | 92 | 1 |
| SARS-CoV | ORF1b | Forward: CAGAACGCTGTAGCTTCAAAAATCT  Reverse: TCAGAACCCTGTGATGAATCAACAG  Probe: (FAM) TCTGCGTAGGCAATCC | 68 | 2 |
| *Burkholderia pseudomallei* | TTS1-orf2 | Forward: CGTCTCTATACTGTCGAGCAATCG  Reverse: CGTGCACACCGGTCAGTATC  Probe:(FAM)CCGGAATCTGGATCACCACCACTTTCC | 115 | 3 |
| *Coxiella burnetii* | IS1111 | Forward: GTCTTAAGGTGGGCTGCGTG  Reverse: CCCCGAATCTCATTGATCAGC  Probe: (FAM)AGCGAACCATTGGTATCGGACGTTTATGG | 295 | 4 |
| *Bacillus anthracis* | pX01 | Forward: CATTAAAGTTTTGGCCTGTATAGTCAA  Reverse: GGATTTGCAGAAGGAATGGAAA  Probe: (FAM) CTGCCACCCTTCG | 67 | 5 |
|  | pX02 | Forward: CGCTGGCGCTTCAATTCT  Reverse: AGAGATGACAAAGCAAGGGATGA  Probe: (FAM) CCTGCTTTCACTGCTT | 61 |  |
| *Francisella tularensis* | ISFtu2F | Forward: TTGGTAGATCAGTTGGTGGGATAAC  Reverse: TGAGTTTTACCTTCTGACAACAATATTTC  Probe:(FAM)AAAATCCATGCTATGACTGATGCTTTAGGTAATCCA | 97 | 6 |
| *Yersinia pestis* | pPCP-1 | Forward: GAAAGGAGTGCGGGTAATAGGTT  Reverse: CCTGCAAGTCCAATATATGGCATA  Probe: (FAM) TAACCAGCGCTTTTC | 63 | 7 |

**Results**

According to MIQE guidelines, specificity and sensitivity (linear dynamic range, amplification efficiency and limit of detection) must be determined for validation.^8^ The primers and probes used were according to published protocols and therefore specificity of assays were not determined. The regression coefficients (R^2^) for all assays were more than 0.99 and the amplification efficiency rates were between 93.1% to 107.9%, which are within the desired range of 90-110% (Table 2). The limits of detection for all assays were between <10 copies/reaction to <50 copies/reaction. All RT-qPCR assays and qPCR assays were well-validated.

**Table 2.** Validation of RT-qPCR and qPCR assays.

| **Pathogens** | **Target gene** | **R^2^** | **Amplification efficiency** | **Limit of detection (LoD)** |
| --- | --- | --- | --- | --- |
| MERS-CoV | UpE | 0.99 | 104.07 | < 25 copies/reaction |
| SARS-CoV | ORF1b | 0.99 | 102.64 | < 25 copies/reaction |
| *B. pseudomallei* | TTS1-ORF2 | 0.99 | 103.56 | < 10 copies/reaction |
| *C. burnetii* | IS1111 | 0.99 | 93.12 | < 10 copies/reaction |
| *B. anthracis* | pXO1  pXO2 | 0.99  0.99 | 94.34  94.58 | < 50 copies/reaction  < 50 copies/reaction |
| *F. tularensis* | ISFtu2 | 0.99 | 94.27 | < 10 copies/reaction |
| *Y. pestis* | pPCP-1 | 0.99 | 91.42 | < 50 copies/reaction |

**References**

1. Corman VM, Eckerie I, Bleicker T, et al. Detection of a novel human coronavirus by real-time reverse-transcription polymerase chain reaction. *Euro Surveill.* 2012;17:20285. doi: 10.2807/ese.17.39.20285-en

2. Poon LLM, Chan KH, Wong OK, et al. Detection of SARS coronavirus in patients with severe acute respiratory syndrome by conventional and real-time quantitative reverse transcription-PCR assays. *Clin Chem.* 2004;50:67-72. doi: 10.1373/clinchem.2003.023663

3. Novak RT, Glass MB, Gee JE, et al. Development and evaluation of a real-time PCR assay targeting the type III secretion system of *Burkholderia pseudomallei*. *J Clin Microbiol.* 2006;44:85-90. doi: 10.1128/JCM.44.1.85-90.2006

4. Klee SR, Tyczka J, Ellerbrok H, et al. Highly sensitive real-time PCR for specific detection and quantification of *Coxiella burnetii*. *BMC Microbiol.* 2006;6:1-8. doi: 10.1186/1471-2180-6-2

5. Parsons TM, Cox V, Essex-Lopresti A, et al. Development of three real-time PCR assays to detect *Bacillus anthracis* and assessment of diagnostic utility. *J Bioterr Biodef* 2013;S3:009. doi: 10.4172/2157-2526.S3-009

6. Versage JL, Severin DDM, Chu MC, Petersen JM. Development of a multitarget real-time Taqman PCR assay for enhanced detection of *Francisella tularensis* in complex specimens. *J Clin Microbiol.* 2003;41:5492-9. doi: 10.1128/JCM.41.12.5492-5499.2003

7. Riehm JM, Rahalison L, Scholz HC, et al. Detection of *Yersinia pestis* using real time PCR in patients with suspected bubonic plague. *Mol Cell Probes.* 2011;25:8-12. doi: 10.1016/j.mcp.2010.09.002

8. Bustin SA, Benes V, Garson JA, et al. The MIQE guidelines: Minimum information for publication of quantitative real-time PCR experiments. *Clin Chem.* 2009;55:1-12. doi: 10.1373/clinchem.2008.112797
