# Supplementary material for: Rhinovirus/enterovirus was the most common respiratory virus detected in adults with severe acute respiratory infections pre-COVID-19 in Kuala Lumpur, Malaysia
Source: PLoS One. 2022 Sep 2;17(9):e0273697. doi: 10.1371/journal.pone.0273697 (PMC9439195; doi:10.1371/journal.pone.0273697)
Supplement: S1 Table — (DOCX) [file pone.0273697.s002.docx]

**S1 Table.** Primers used for genotyping rhinovirus/enterovirus.

|  | **Primer** | **Sequence (5’ -3’)** | **Amplicon size (bp)** | **Reference** |
| --- | --- | --- | --- | --- |
| **Enterovirus typing**  **(VP1)** | AN32  AN33  AN34  AN35 | GTYTGCCA  GAYTGCCA  CCRTCRTA  RCTYTGCCA | Not applicable | 1 |
|  | 222  224 | CICCIGGIGGIAYRWACAT  GCIATGYTIGGIACICAYRT | 762 |  |
|  | AN88  AN89 | CCAGCACTGACAGCAGYNGARAYNGG  TACTGGACCACCTGGNGGNAYRWACAT | 348 – 39 |  |
| **Rhinovirus typing (VP4/VP2)** | DK001  SRHI1  SRHI2 | CAAGCACTTCTGTTTCCC  GCATCIGGYARYTTCCACCACCANCC  GGGACCAACTACTTTGGGTGTCCGTGT | 913bp  950bp | 2 |

**References**

1. Nix WA, Oberste MS, Pallansch MA. Sensitive, seminested PCR amplification of VP1 sequences for direct identification of all enterovirus serotypes from original clinical specimens. *J Clin Microbiol.* 2006;44:2698-704. doi: 10.1128/JCM.00542-06

2. Fall A, Dia N, Kebe O, Sarr FD, Kiori DE, Cisse EHAK, et al. Enteroviruses and rhinoviruses: molecular epidemiology of the most influenza-like illness associated viruses in Senegal. *Am J Trop Med Hyg.* 2016;95:339-47. doi: 10.4269/ajtmh.15-0799
