# Supplementary material for: Rhinovirus/enterovirus was the most common respiratory virus detected in adults with severe acute respiratory infections pre-COVID-19 in Kuala Lumpur, Malaysia
Source: PLoS One. 2022 Sep 2;17(9):e0273697. doi: 10.1371/journal.pone.0273697 (PMC9439195; doi:10.1371/journal.pone.0273697)
Supplement: Attachment — Submitted filename: Response to reviewers.pdf [file pone.0273697.s003.pdf]

RESPONSES TO REVIEWERS

PONE-D-22-15536
Rhinovirus/enterovirus was the most common respiratory virus detected in adults with severe acute respiratory infections pre-COVID-19 in Kuala Lumpur, Malaysia

We thank the reviewers for their time and efforts in reviewing this paper.

Reviewer #1:
The aim of the study was to detect viral etiologies in adults admitted with SARI in Kuala Lumpur, Malaysia in the 2 years before COVID-19, using molecular assays and metagenomics analysis. Samples of 489 patients were evaluated by a commercial multiplex nucleic acid assay (Luminex assay). In 57.1% of the patients one or more pathogens were detected. Rhinovirus/enterovirus (RV/EV) was the most prevalent agent that detected in nearly half of the samples, followed by influenza virus. In 105 Luminex negative samples were evaluated by a viral metagenomic analysis. A positive result was detected in 2% samples. In addition, factors related to increased risk of critical disease were studied. RV/EV isolates were characterized by sequencing.

This is a study providing information regarding viral pathogens causing SARI in pre-COVID-19 era in a single center. It also reports the value of further investigation with metagenomics analysis to detect the pathogen in primer assay negative samples. Although results are not unique, the manuscript is well written, methods are clearly described and laboratory results are combined with patients clinical details.

Thank you for your positive review.

Reviewer #2:
This manuscript is an epidemiological study investigating the causative virus of adult severe respiratory infections in Malaysia. I don't think there are any particular problems in considering the methods and results of this research.
However, this study is an epidemiological study in a very limited area of Malaysia and is not considered to reflect the whole of Malaysia or Asia.

This is an important limitation which we have acknowledged in the discussion (p23, lines 371-373):
"It involved a single hospital, and only 105/218 (48.2%) negative samples underwent viral metagenomics analysis. Broader and more extensive surveillance studies are needed for more nationally representative data."

In addition, this study was performed before the COVID-19 pandemic, and if possible, it would be better to investigate what kind of changes have occurred since the COVID-19 pandemic.

This is an important point, as other respiratory viruses have been widely shown to have drastically reduced during the pandemic. We have separately published a similar study carried out in the early months of the pandemic which supports this, and have cited this in the discussion of the limitations of our study (p23, lines 375-377):

"This study was conducted before the COVID-19 pandemic, which was associated with declines in other respiratory viruses globally, including at our centre [41,42]."
(Ref 41 is our study, and can be found at https://doi.org/10.1016/j.jcv.2021.105000)

Therefore, I don't think it will be cited by many people at this point. Or, its scientific value is not high.

While the data is not novel, we believe, as we have written in the discussion, that "it provides important baseline data of circulating respiratory viruses in a tropical country." This data is certainly lacking in adults in Malaysia and due to the cost of multiplex PCR/NGS and competing demands on limited diagnostic resources, is unlikely to be widely collected apart from sporadic, well-funded research projects or private hospitals.

Furthermore, it is evident that as COVID-19 numbers have relatively waned this year, there has been a resurgence of respiratory viruses which were much reduced during the pandemic, such as influenza, RSV and adenovirus. Therefore, the post-pandemic virus circulation patterns are more likely to resemble the pre-pandemic circulation of multiple viruses. This adds to the importance of our baseline data for 2017-2019.
